# Successful renal transplantation after two separate urinary tract malignancies

**DOI:** 10.4103/0970-1591.40625

**Published:** 2008

**Authors:** Rohit Joshi, Kim Mammen, Basant Pawar

**Affiliations:** Medical Oncology, Royal Adelaide Hospital, Adelaide SA 5000, Australia; 1 Department of Urology, Christian Medical College, Ludhiana - 141 008, India; 2 Department of Nephrology, Christian Medical College, Ludhiana - 141 008, India

**Keywords:** Renal cell carcinoma, transitional cell carcinoma, transplantation

## Abstract

A patient who was treated for renal cell carcinoma and transitional cell carcinoma, later presented with end stage renal disease. He was managed with hemodialysis and later underwent successful renal transplantation. There was no evidence of tumor recurrence nearly nine years post-renal transplantation.

## INTRODUCTION

The case of a patient who was treated for renal cell carcinoma and transitional cell carcinoma with no evidence of recurrence having undergone successful renal transplantation is being reported.

## CASE REPORT

A 37-year-old male underwent right-sided radical nephrectomy for organ-confined renal cell carcinoma (RCC) measuring 8 × 7 cm involving the lower and middle third of the kidney. The histology was clear cell carcinoma (Fuhrman nuclear Grade 1) with no involvement of the regional lymph nodes (T1N0M0). Six years later, he presented with macroscopic hematuria. Cystoscopy revealed a 2 cm × 2 cm Grade II transitional cell carcinoma (TCC) on the anterior wall of the urinary bladder that was treated with transurethral resection of bladder tumor (TURBT) with adjuvant intravesical BCG immunotherapy (pathological staging was pT1G2). One hundred and twenty mg of BCG was given weekly as an intravesical instillation for six weeks as induction therapy. No maintenance therapy was given. Regular urine cytological examination and check cystoscopy revealed no recurrence and progression-free patient.

Six months after the TURBT, he was admitted with chronic renal failure (CRF), due to an undetermined etiology in the setting of longstanding diabetes mellitus and prolonged use of non-steroidal anti-inflammatory medications. His serum creatinine was 2.4 mg/dl and blood urea 40 mg/dl. Urine analysis showed 3 +++ proteinuria with 8-10 red blood cells and 10-12 pus cells. His renal functions progressively deteriorated. Four years later, he developed end stage renal disease and was started on maintenance hemodialysis. He underwent a living related renal transplantation, which was 10 and four years after RCC and TCC were treated respectively. Immunosuppression comprised prednisolone, azathioprine and cyclosporine. He remains well with regular clinical review, urine cytology, ultrasound and check cystoscopy examinations. His renal functions are normal and there is no evidence of local or systemic recurrence 19 years post-excision of RCC and nearly nine years post-renal transplantation.

## DISCUSSION

Transplantation with its attendant immunosuppression may predispose patients to tumor recurrence;[1] however, most of the information regarding the outcome in patients with treated malignancy undergoing organ transplantation remains anecdotal.

Therapeutic options for patients with bilateral renal cell cancer or disease in a solitary kidney are limited to partial nephrectomy or bilateral radical nephrectomy with subsequent dialysis and transplantation. Few reports discuss the long-term survival of patients receiving chronic dialysis or after renal transplantation. Information regarding the long-term prognosis for these patients is important when deciding on the appropriate treatment. The recommended waiting period for renal transplantation after bilateral nephrectomy or removal of a solitary kidney, is two years. During this time the patient receives dialysis, before proceeding with transplantation, provided there is no evidence of recurrence of tumor.[2] Renal cell carcinoma and TCC in the transplant population behaves aggressively and warrants careful attention before and after renal transplantation.[3,4]

Certain patients treated for cancer may be candidates for kidney transplantation, even if the risk of cancer is higher in transplant recipients. Moreover, kidney transplantation without the recommended two-year waiting period on maintenance dialysis could be proposed in early-stage low-grade RCC.[5] However, no long-term data is available. Renal transplantation can provide satisfactory replacement therapy for patients with end stage renal disease with von Hippel-Lindau Disease (VHLD) with treated RCC.[1]

This patient had RCC and TCC, which were adequately treated and there was no evidence of recurrence. We have been unable to find a case of two tumors of the urinary tract that was successfully treated and then underwent renal transplantation. We feel that renal transplantation can be offered to even those patients who have more than one malignancy in the urinary tract, provided that both have been successfully treated and there being no evidence of recurrence.
